# Vaccination against IL-33 Inhibits Airway Hyperresponsiveness and Inflammation in a House Dust Mite Model of Asthma

**DOI:** 10.1371/journal.pone.0133774

**Published:** 2015-07-27

**Authors:** Ying Lei, Vamsi Boinapally, Anna Zoltowska, Mikael Adner, Lars Hellman, Gunnar Nilsson

**Affiliations:** 1 Department of Medicine, Clinical Immunology and Allergy Unit, Karolinska Institutet and Karolinska University Hospital, Stockholm, Sweden; 2 Department of Cell and Molecular Biology, Uppsala University, Uppsala, Sweden; 3 Institute of Environmental Medicine, Karolinska Institutet, Stockholm, Sweden; 4 Center for Allergy Research, Karolinska Institutet, Stockholm, Sweden; 5 Department of Pharmacy, The Second Affiliated Hospital, School of Medicine, Xi'an Jiaotong University, Xi'an, China; Academic Medical Centre/University of Amsterdam, NETHERLANDS

## Abstract

In several clinical and experimental studies IL-33 and its receptor have been found to play important roles in the development of asthma and allergic airway inflammation. We evaluated the effects of vaccination against IL-33 in a mouse model of airway inflammation induced by house dust mite (HDM) allergen. Balb/c mice received the IL-33 vaccine subcutaneously, followed by intranasal administration of HDM for up to six weeks. Vaccination against IL-33 induced high titers of specific anti-IL-33 IgG antibodies that inhibited HDM-induced airway hyperresponsiveness (AHR) in the conducting airways and tissue damping. The vaccination also attenuated the HDM-induced elevation in the numbers of eosinophils in bronchoalveolar lavage fluid (BALF) and suppressed the accumulation of inflammatory cells in the airways. Furthermore, the levels of IL-17A, IL-25, IL-33 and TSLP in lung tissue homogenates were reduced by vaccination against IL-33. These observations demonstrate that vaccination against IL-33 inhibits HDM-induced development of AHR, airway inflammation and production of inflammatory cytokines. The results also indicate an important role of IL-33 in the regulation of AHR of the distal lung compartments. Thus, administration of such a vaccine is potentially an effective therapeutic tool for treating allergic asthma.

## Introduction

Asthma, a common airway disease affecting many people in developed countries requiring considerable health care costs, exhibits a wide variety of phenotypes, with limitation of airflow and bronchial hyperresponsiveness [1]. This condition is often associated with airway inflammation and remodeling, where the airway epithelium acts as an important regulator of inflammatory responses to exogenous agents such as allergens, viruses and pollutants. The physical barrier by the epithelium appears to be defective in patients with asthma, allowing easier penetration of inhaled agents. Accordingly it has been proposed that one major defect characteristics of asthma are the aberrant interactions between damaged epithelial cells and underlying structural and resident cells, which promotes chronic inflammation and remodeling [2].

At present asthma is treated primarily by daily administration of β_2_-adrenoceptor agonists, corticosteroids and anti-leukotrienes. Many patients may fail to comply with the need for daily use and, moreover, respond poorly to corticosteroids. Therefore, novel, effective treatment regimens are required and recently increasing attention has been focused on interfering with the inflammatory process, as well as on treating steroid-insensitive asthma. In an attempt to develop such new therapies for asthma, as well as for other severe forms of allergy such as atopic dermatitis, we have explored the possibility of employing traditional vaccine technology, with modified endogenous molecules, to modulate the *in vivo* levels of key regulatory molecules [3].

In past years our understanding of the initial regulation of inflammatory responses, including the function of barriers, has advanced considerably. Several cytokines are now known to be key regulators of events that can lead to the development of airway hyperresponsiveness (AHR) and chronic inflammation, including IL-18, IL-25, IL-33 and thymic stromal lymphopoietin (TSLP), all of which are released by epithelial cells [4, 5]. The potential involvement of IL-33 in the etiology of asthma has attracted considerable attention as a consequence of recent large-scale genome-wide association and polymorphism studies that link the genes for IL-33 (*IL33*) and its receptor (*IL1RL1*) to asthma susceptibility [6–8]. Moreover, a recent report documents a strong association between polymorphisms in IL-33 and hospitalization for asthma, an indicator of disease severity [9]. Furthermore, IL-33 is expressed at elevated levels in the bronchial epithelium cells and airway smooth muscle cells of asthmatic patients [10, 11] and is recovered at increased levels in bronchoalveolar lavage fluids (BALF) following an allergen challenge [12]. In addition, several animal models indicate that IL-33 and its receptor play an important role in the development of asthma and allergic airway inflammation. For example, in murine models of asthma involving sensitization by ovalbumin (OVA) or house dust mite (HDM) the level of IL-33 in lung tissue is elevated [13, 14]. Thus, the epithelial-derived cytokine IL-33 appears to act on a variety of inflammatory and structural cell types to promote the development of asthma [15].

To examine the potential suitability of IL-33 as a target for vaccination against allergic asthma, we have developed a fusion protein vaccine against this cytokine. The present investigation was designed to evaluate the effects of this vaccine in a murine HDM-induced model of asthma. These animals demonstrate many of the pathological features characteristic of persistent asthma in humans, including eosinophilic airway inflammation, hypersecretion of mucus, and AHR in response to methacholine (MCh)[16]. We found that this vaccine elicits high titers of neutralizing anti-IL-33 antibodies that are sustained and exert a marked impact on airway resistance, eosinophil infiltration and cytokine expression. Thus, this vaccine appears to be a promising candidate for future treatment of various inflammatory conditions mediated by IL-33.

## Materials and Methods

### Preparation of the vaccine antigen, carrier protein and ELISA reagents

**Fig 1:**
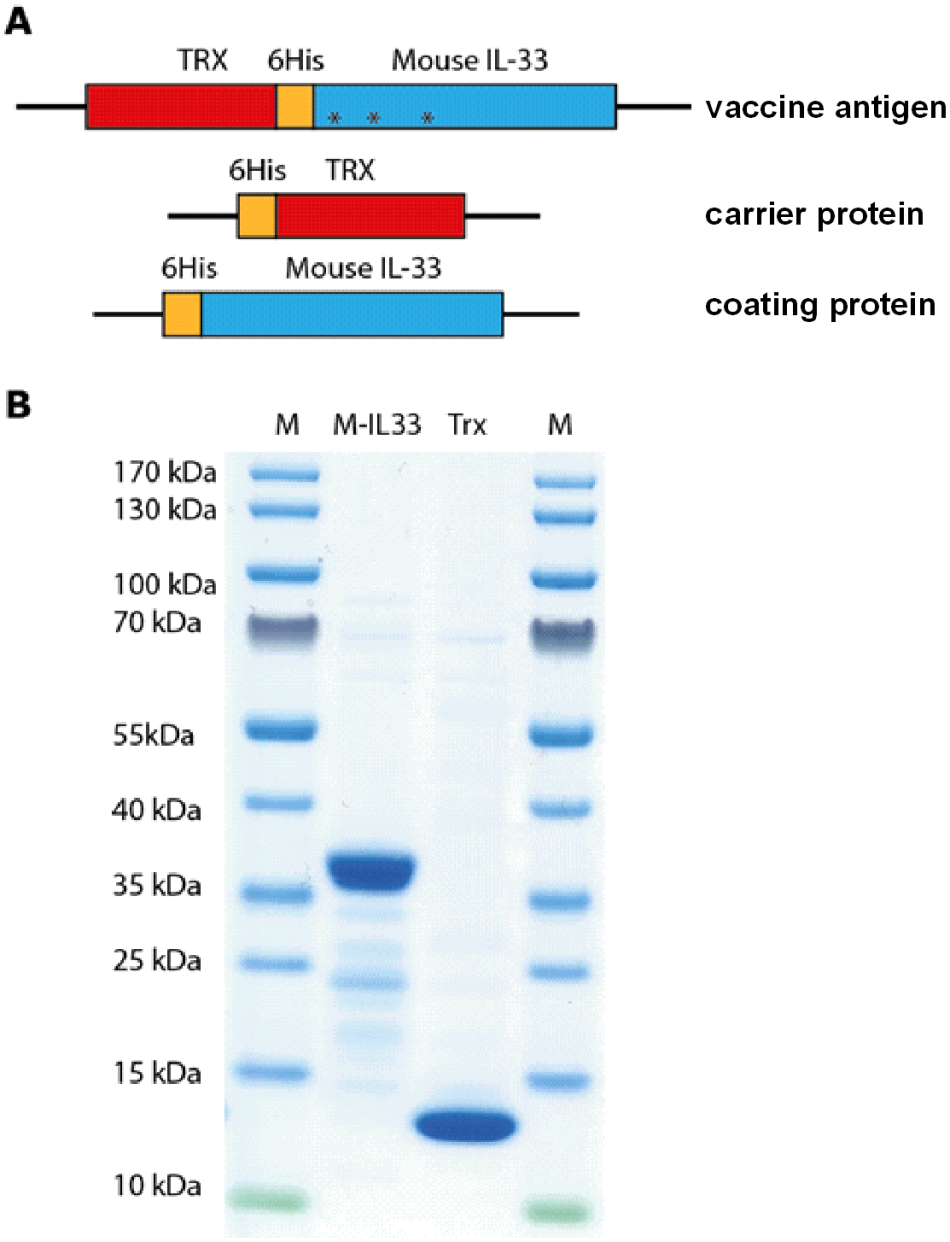
The recombinant proteins employed in the present investigation. (A) Schematic structures of the vaccine antigen (Trx-His-IL-33), the carrier protein (His-Trx) and the His-tagged IL-33 protein used for coating the ELISA plates. (B) SDS-PAGE gel analysis of the vaccine antigen and the carrier proteins used for immunization. Trx, thioredoxin; 6His, six-histidine tag.

The coding region of the gene for mouse IL-33 was mutated in three positions in order to reduce its receptor binding capacity. The codon-optimized construct encoding IL-33 and the coding region of the *Escherichia*.*coli* (*E*.*coli*) gene for thioredoxin (Trx) were subcloned into the pET21 vector to obtain a His_6_-tag fusion protein that was then expressed in *E*.*coli* Rosetta gami (Novagen Merck Darmstadt, Germany). Following ultrasonic lysis this protein was purified by affinity chromatography on Ni-NTA agarose beads (Qiagen, Hilden, Germany). A schematic representation of the constructs employed is depicted in Fig 1.

### Ethics statements

This study was conducted with the approval of the Regional Committee of Animal Experimentation Ethics at Karolinska institutet (Stockholm, Sweden, permit number: N443/11). All surgery was performed under sodium pentobarbital anesthesia, and all efforts were made to minimize suffering.

### Immunization and intranasal treatment of mice with HDM allergen

**Fig 2:**
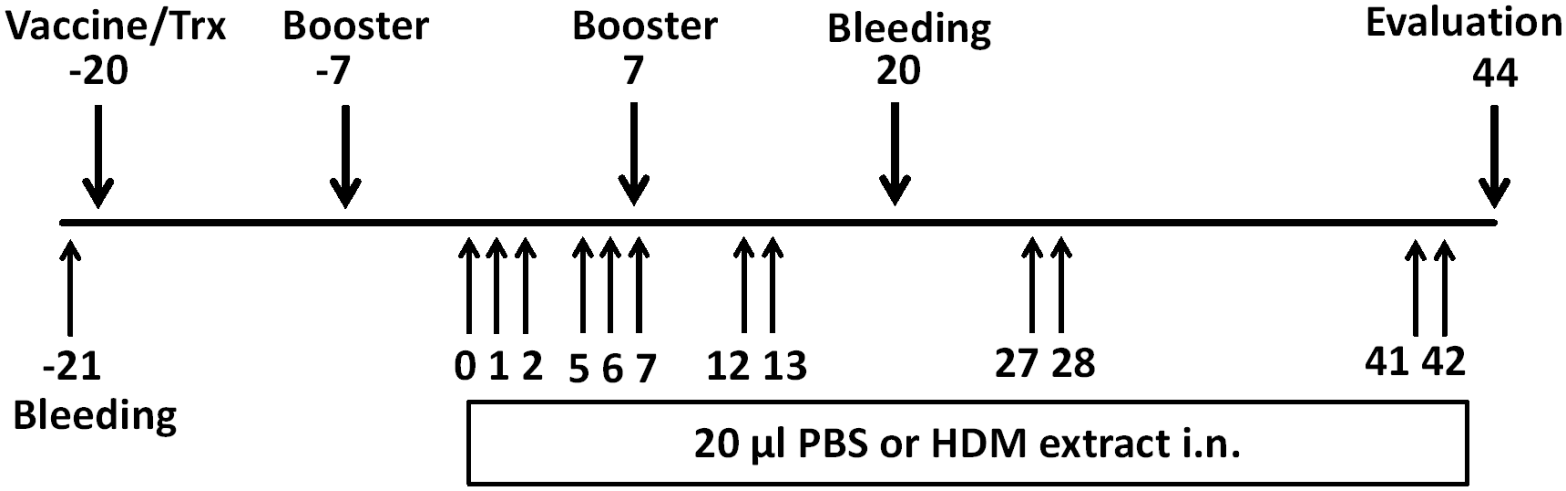
Protocol for the vaccination and intranasal exposure to house dust mite. Blood was collected on day -21 to detect the baseline of anti-IL-33 IgG level. Vaccine (100 μg of IL-33 recombinant protein or carrier protein) was administrated subcutaneously from day-20, three times at a two-week interval. On days 0 (one week prior to the final immunization), 1, 2, 5, 6, 7, 12, 13, 27, 28, 41 and 42, mice were challenged by intranasal administration of HDM extract (25 μg) in 20 μl PBS or equal volume PBS On day 44 lung physiology assessment was performed and samples were taken for analysis. HDM, house dust mite; Trx, thioredoxin; i.n., intranasal; PBS, phosphate buffered saline.

Female BALB/c Mice (Charles River, Sluzfeld, Germany, 8–10 weeks) were immunized subcutaneously with 100 μl (100 μg) of IL-33 recombinant protein or carrier protein (thioredoxin) alone three times at two-week intervals (Fig 2). Prior to injection, these proteins were mixed with Montanide ISA 720 (Seppic, France) and 50 μg of a phosphorothioate stabilized CpG oligonucleotide 1826 [17] as adjuvants, and the resulting mixture emulsified. Skin reactivity to the IL-33 vaccine was not observed. One week prior to the final immunization, a 42-day protocol designed to produce chronic lung inflammation by repeated intranasal exposure of the mice (under anesthesia with isoflurane) to HDM (ALK-Abello, Denmark) was initiated (Fig 2), mice were challenged by intranasal administration of HDM extract (25 μg) in 20 μl PBS or equal volume PBS. Thereafter, mice were divided into three groups: IL-33 vaccine plus HDM exposure group, carrier protein plus HDM exposure group and IL-33 vaccine plus PBS exposure group.

### Measurement of airway hyperresponsiveness

Pulmonary mechanics following intravenous injections of increasing concentrations of methacoline (MCh) (Sigma-Aldrich, Sweden) were assessed in a flexiVent apparatus (Scireq, Montreal, Canada), as described previously [18, 19]. Mice were anesthetized i.p. with penthobarbital (70–90 mg·kg^-1^ body weight; Apoteket Produktion & Laboratorier AB, Stockholm, Sweden), tracheotomised with a blunted 18-gauge cannula and ventilated (flexiVent, SCIREQ, Montreal, Canada). Using the forced-oscillation technique, ventilation was performed at 2.5 Hz in a quasi-sinusoidal fashion in order to generate a sinusoidal pressure waveform during lung inflation. Utilizing the forced-oscillation technique, pulmonary resistance was determined assuming a single-compartment linear model. Resistance in conducting airways (*Rn*), tissue damping (*G*) and tissue elastance (*H*) were evaluated by assuming a constant-phase model. The flexiVent software calculates a coefficient of determination (COD) to establish the goodness of fit of this model to the data. Any data point with a COD < 0.9 is automatically rejected.

### Tissue collection and preparation

Bronchoalveolar lavage (BAL) was performed after airway hyperresponsiveness measurements when the animal was still under anesthesia. A total volume of 0.8 ml cold PBS was used to lavage the lungs three times. Blood was collected by cardiac puncture with a 20-gauge needle, and serum was prepared through centrifugation in serum tubes (Sarstedt, Helsingborg, Sweden) and stored at -20°C for further analyses. The mice were euthanized with cervical dislocation. The left lung lobe was removed and fixed in 4% formaldehyde (Histolab, Stockholm, Sweden) overnight. The superior and middle lobes of the right lung were snap frozen on dry ice and stored at -80°C until further processing. Protein was extracted from the snap-frozen samples of pulmonary tissue with a cell lysis kit (Bio-plexTM Cell Lysis kit, Bio-Rad) supplemented with proteinase inhibitors (Sigma-Aldrich).

### Measurements of anti-IL-33 and anti-HDM antibody responses

Blood samples were taken at days -21, 20 and 44. To assay serum IgG specific for IL-33, plates were coated with mouse recombinant IL-33 (Fig 1A) and incubated with serum diluted 2500-fold and thereafter with alkaline phosphatase-conjugated goat anti-mouse IgG (Jackson ImmunoResearch Laboratories, Inc., PA, USA). For the measurement of HDM-specific IgG and IgE antibodies, plates were coated with the HDM preparation (50 μg protein/ml) [20] and subsequently incubated with serum (diluted 1:5), followed by incubation with alkaline phosphatase-conjugated goat anti-mouse IgG and IgE.

### Assessment of lung histology

Following appropriate preparation, 5 μm-thick paraffin sections of the left lung were stained (hematoxylin and eosin; Histolab) to detect infiltration of inflammatory cells. Pulmonary inflammation was assessed in a blinded manner by semi-quantitative evaluation of the infiltration: 0 = no; 1 = slight; 2 = moderate; 3 = abundant, as reported elsewhere [18]. In sections stained with alcian blue-periodic acid–Schiff (AB/PAS), goblet cells in the airway epithelium were counted employing a semi-quantitative scoring system: 0 = < 5% goblet cells; 1 = 5–25%; 2 = 25–50%; 3 = 50–75%; 4 = > 75% [21].

### Quantification of cytokines in lung homogenates

In extracts of snap-frozen samples of pulmonary tissue prepared with a cell lysis kit (Bio-plex^TM^ Cell Lysis kit, Bio-Rad) supplemented with proteinase inhibitors (Sigma-Aldrich) the levels of IL-5, -13, -17A, -25, -33, and TSLP were quantified with ELISA kits (eBioscience).

### Statistical analysis

One-way or two-way ANOVA, the χ2 test, and Bonferroni’s post-hoc test were applied for statistical analyses (Graph Pad Prism 5, La Jolla, CA). A *P* value of ≤0.05 was considered statistically significant. Ten animals were included in each group. The actual animal number in each graph has been indicated in the figure legends.

## Results

### Preparation of the vaccine antigen and selection of adjuvant

To develop a vaccine that is both safe and can break self-tolerance against IL-33 effectively we analyzed fusion partners and mutations that results in a vaccine antigen that is soluble; lacks cytokine activity; but retains most of its B cell epitopes. Three mutations were made in IL-33 to reduce its receptor binding capacity. As no detailed mutational analysis has been made on IL-33 the mutations were based on similarity to IL-18 where a very detailed alanine scanning analysis has been performed. The inactivity was then tested by subcutaneous injection of 100 ug of the mutated protein. No skin reaction was observed showing the lack of cytokine activity (data not shown). The mutated IL-33 was fused to thioredoxin (Trx) a small, highly soluble bacterial protein (Fig 1A), and the fusion protein was expressed in a bacterial expression system (Fig 1B). The second important factor for successful vaccination against self-antigens is the adjuvant employed, which consisted here of a combination of Montanide ISA720 and the classical 1826 phosphorothioate-stabilized CpG oligonucleotide. This biodegradable depot adjuvant elicits high titers of anti-self-antibodies, as effectively as the most potent adjuvant known, a combination of Freunds complete and incomplete adjuvant [17, 22, 23]. Since a higher dose of antigen is usually required for successful vaccination against self-antigens than against virus or bacteria, 100 μg of our vaccine antigens was used together with this adjuvant.

### Vaccination against IL-33 elicits specific IgG antibodies

**Fig 3:**
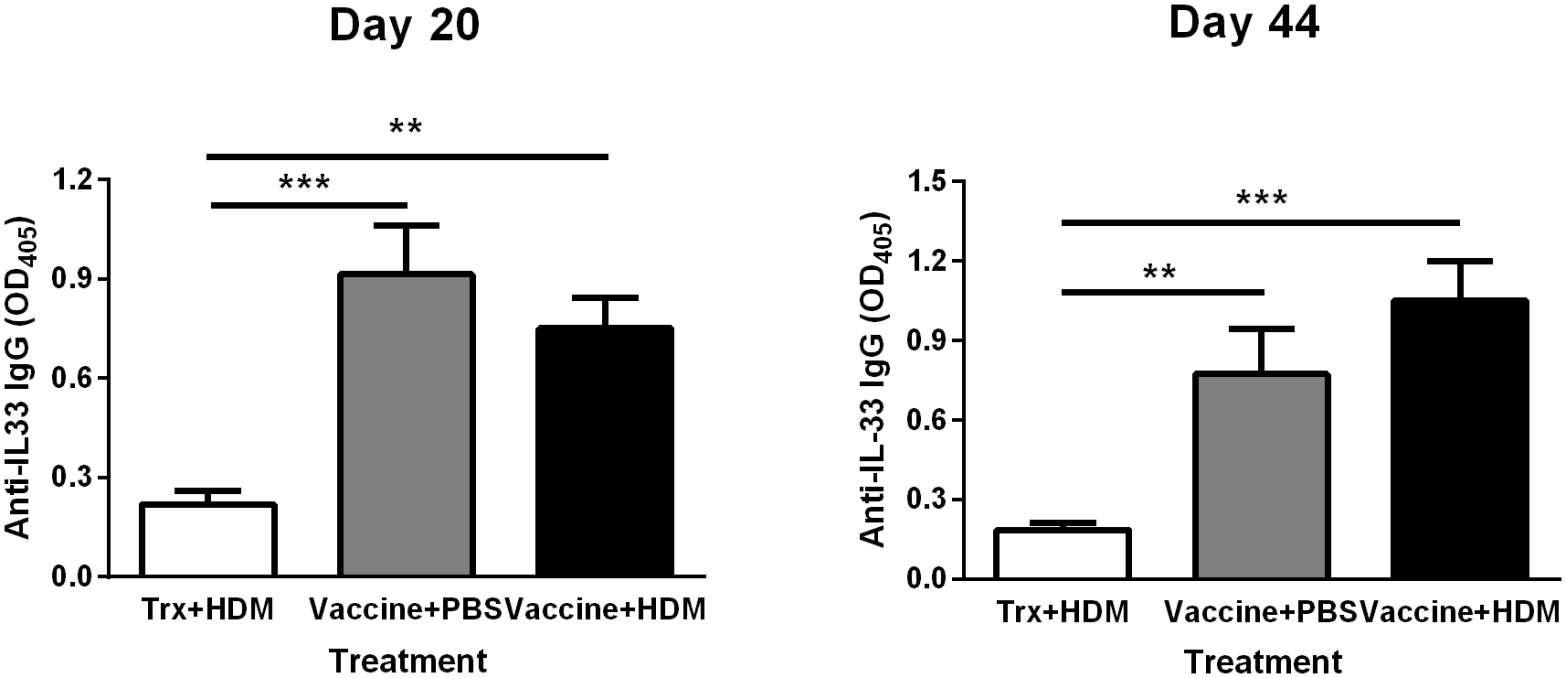
IL-33 vaccine elicits anti-IL33 specific antibodies. Serum samples of anti-IL-33 IgG on days 20 (left) and days 44 (right). All values shown are means ± s.e.m (n = 10 mice in each group on day 20; n = 9 mice in HDM+Vaccine group, n = 10 in PBS+Vaccine group and n = 10 in Trx+HDM group on day 44, missing sample due to failed collection of serum from the animal). ** *P* < 0.01, *** *P* < 0.001 in comparison to the Trx + HDM group. Vacc: IL-33 vaccine, Trx: thioredoxin, HDM: housedust mite, N.D.: not detected.

The ability of the vaccine to evoke an IL-33-specific IgG response was analyzed by ELISA. A bacterial extract was added during dilution of the sera to eliminate interference from antibodies towards contaminating bacterial proteins. A 1:2500 dilution of serum resulted in a high signal/background ratio, revealing that high titers of specific antibodies against IL-33 were obtained as early as days 20 (13 days after the last immunization) and 44 (37 days after the last immunization), while the anti-IL-33 IgG was not detected in serum of naïve mice before the vaccination on day -21 (data not shown). Thus, the recombinant IL-33 vaccine is highly immunogenic (Fig 3).

### Vaccination against IL-33 inhibits the airway hyperresponsiveness induced by HDM

**Fig 4:**
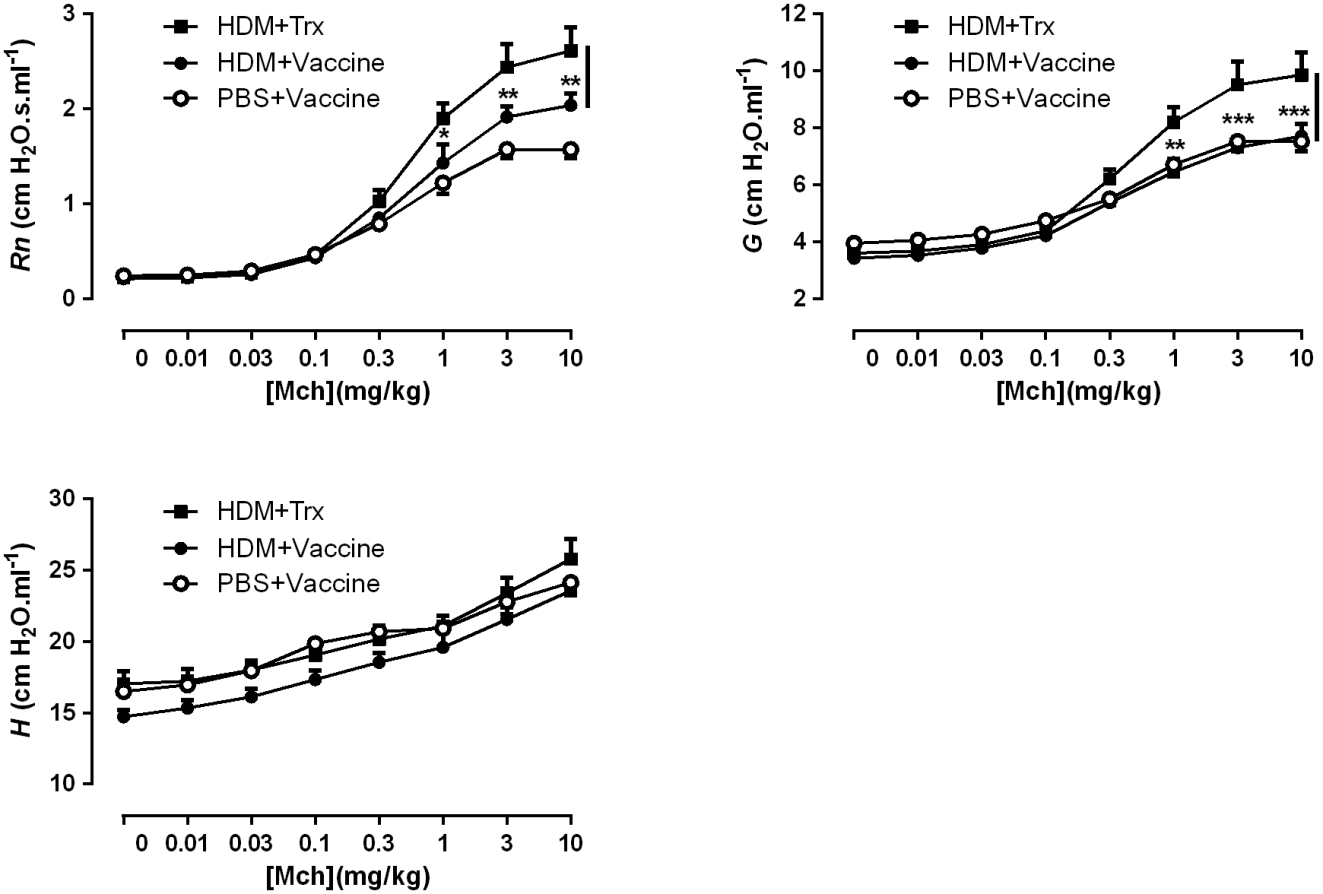
Vaccination against IL-33 diminishes house dust mite-induced airway hyperresponsiveness to methacholine. The resistance of the central airways (*Rn*), pulmonary tissue damping (*G*) and tissue elastance (*H*) to methacholine (Mch) were assessed employing a flexiVent. All values shown are mean ± s.e.m. (n = 8 mice in HDM+Vaccine group, n = 9 mice in Trx+HDM group and n = 6 mice in PBS+Vaccine group, missing samples due to unsuccessful or uncompleted intravenous injection in the animals) *P < 0.05, ** *P* < 0.01 in comparison to the Trx+HDM group. Vacc: IL-33 vaccine, Trx: thioredoxin, HDM: house dust mite.

Using flexiVent measurement of airway hyperresponsivness to increasing i.v. doses of MCh in anesthetized, tracheostomised, and ventilated mice sensitized to HDM we could determine a significant increase in both conducting airways resistance (*Rn*) and tissue damping (*G*) (Fig 4). Vaccination against IL-33 significantly inhibited the resistance in the conducting airways (*Rn*) caused by HDM and totally abolished the HDM-induced effect on the tissue damping (*G*); but had no influence on tissue elastance (*H*) (Fig 4).

### Vaccination against IL-33 reduces the levels of inflammatory cells in bronchoalveolar lavage fluid and suppresses pulmonary inflammation

**Fig 5:**
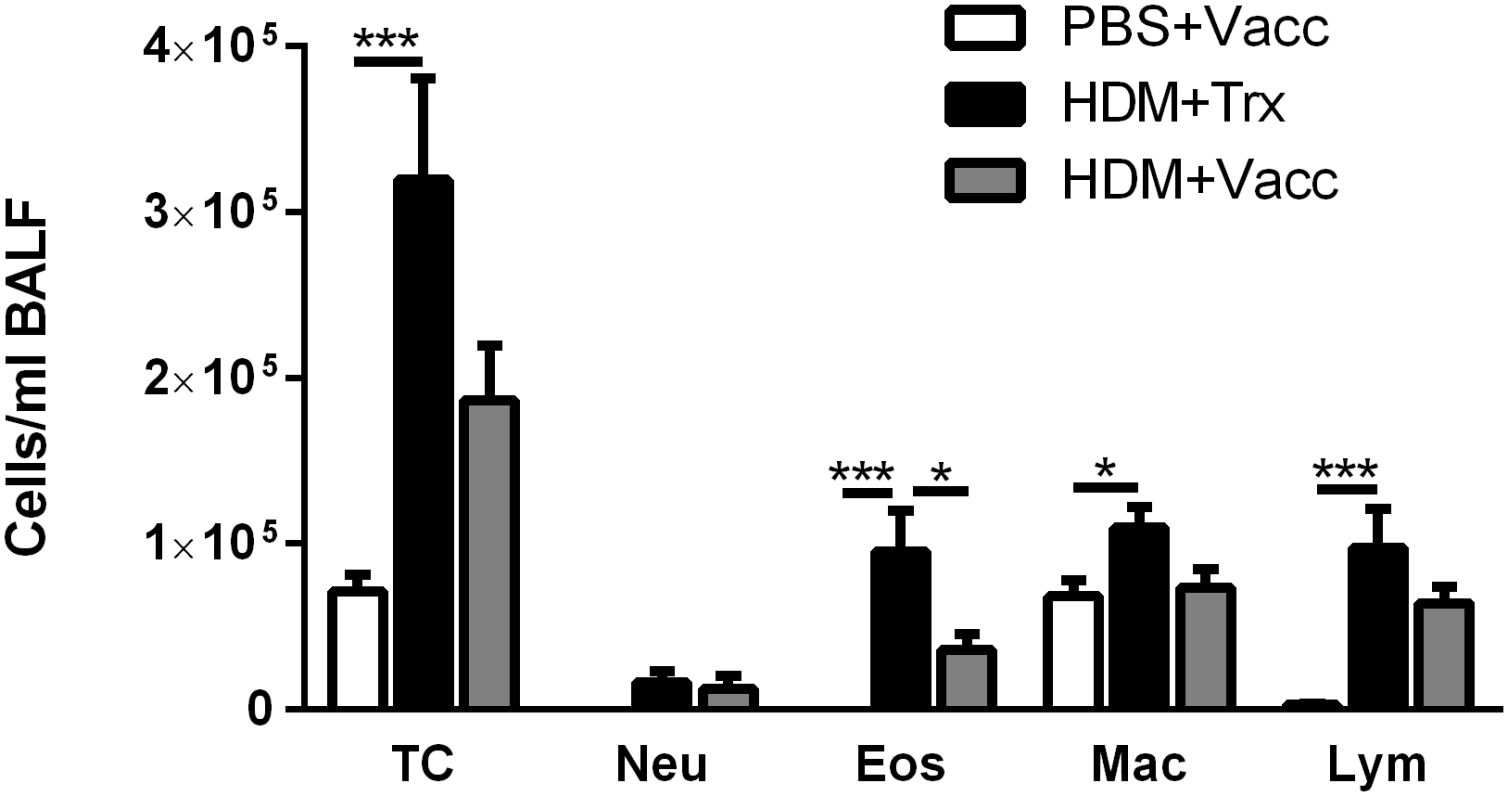
Vaccination of mice against IL-33 attenuates the increase in the number of inflammatory cells present in bronchoalveolar lavage fluids. Total and differential cell counts were performed on cytospin preparations of the BALF. All values shown are mean ± s.e.m. (n = 9 mice in HDM+Vaccine group, n = 10 mice in PBS+Vaccine group and n = 10 mice in Trx+HDM group, missing sample due to failed collection of BALF from the animal). * *P* < 0.05 in comparison to the Trx+HDM group. Vacc: IL-33 vaccine, Trx: thioredoxin, HDM: house dust mite, TC: total cell count, neu: neutrophils, eos: eosinophils, mac: macrophages, lym: lymphocytes.

Sensitization with HDM potently elevated the total number of inflammatory cells in BALF, particularly eosinophils and lymphocytes, but also neutrophils and macrophages (Fig 5). Vaccination against IL-33 significantly reduced the HDM-induced increase in the level of eosinophils in BALF (*P* < 0.05) and tended to attenuate the increases in the total number of inflammatory cells (*P = 0*.*09*) in BALF evoked by HDM (Fig 5). There was no significant effect of vaccination on lymphocyte or macrophages.

**Fig 6:**
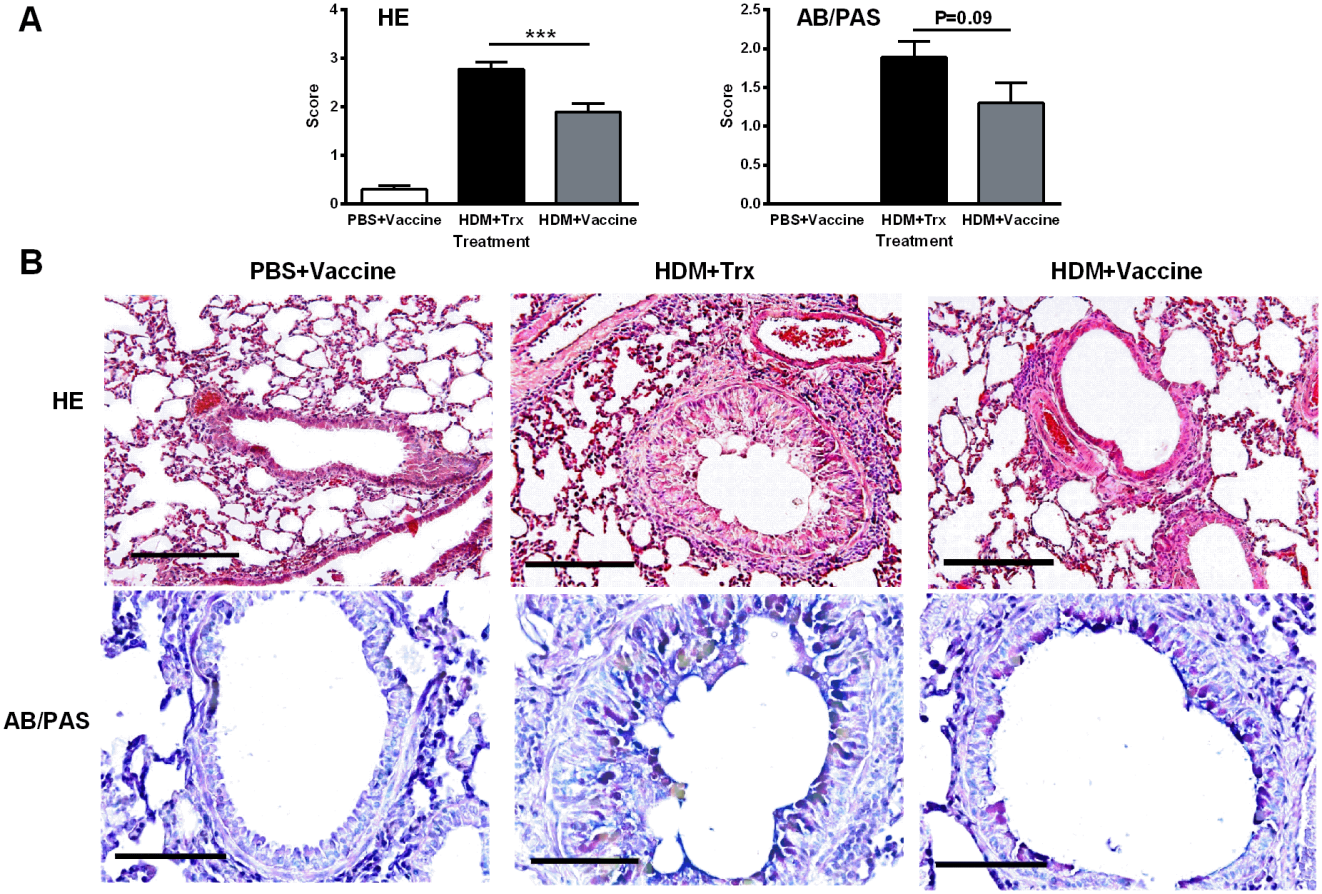
Pulmonary inflammation induced in mice by house dust mite is attenuated by vaccination against IL-33. (A) Semi-quantitative scoring of hematoxylin and eosin (H&E) staining and (B) Alcian blue/periodic acid-Sciff (AB/PAS) staining. (C) Lung sections stained with hematoxylin and eosin, scale bar, 100μm. (D) Lung sections stained with alcian blue/periodic acid-Sciff, mucin-containing cells are periodic acid-Schiff (PAS)-positive (purple colored). Scale bar, 50μm. All values shown are mean ± s.e.m. (n = 10 mice in HDM+Vaccine group, n = 9 mice in Trx+HDM group and n = 10 in PBS+Vaccine group, missing sample due to unsuccessfully proceeding of the lung tissue from the animal). *** *P* < 0.001 in comparison to HDM+Trx group. Vacc: IL-33 vaccine, Trx: thioredoxin, HDM: house dust mite.

Staining with hematoxylin-eosin and alcian blue/periodic acid Schiff revealed that sensitization to HDM evoked a potent inflammatory response, reflected in both perivascular and peribronchial accumulation of inflammatory cells, as well as goblet cell hyperplasia (Fig 6). Vaccination against IL-33 attenuated this accumulation of inflammatory cells (*P* < 0.01); although not statistical significant, the vaccine tended to reduce the epithelial goblet cell hyperplasia (*P = 0*.*09*, Fig 6).

### The induction of HDM-specific IgG is not affected by vaccination

**Fig 7:**
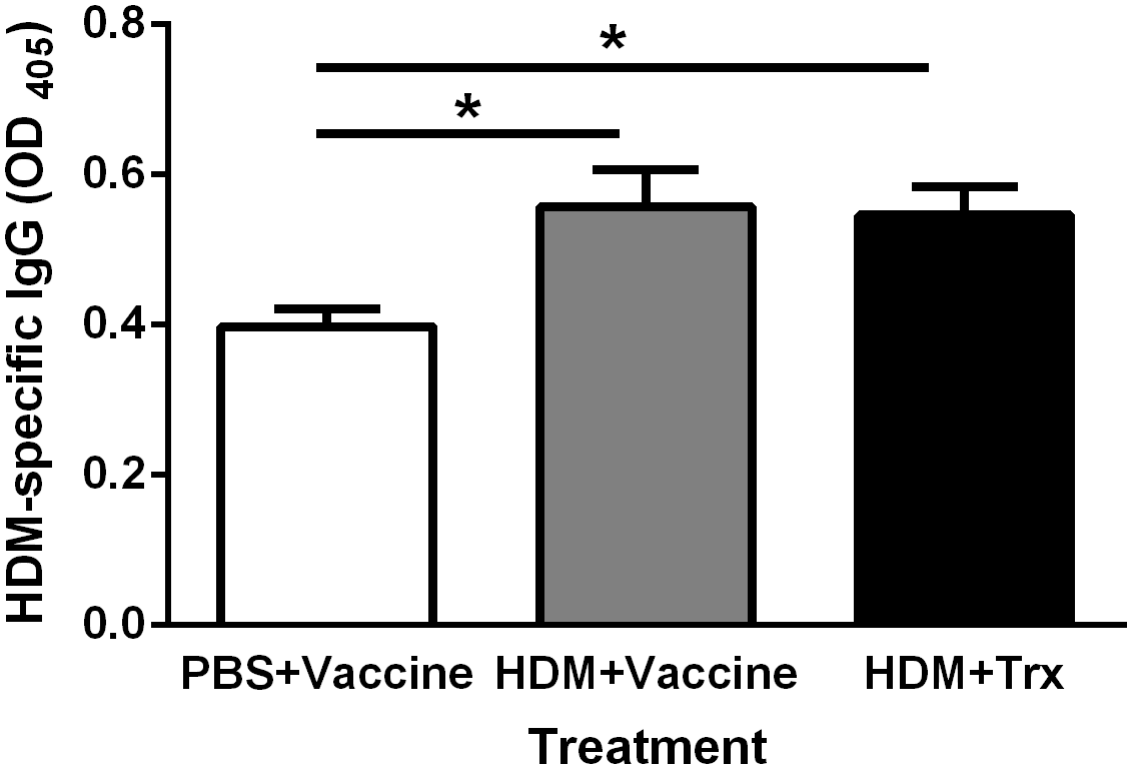
The induction of house dust mite—specific IgG antibodies in mice sensitized to house dust mite is unaffected by vaccination against IL-33. HDM- specific IgG antibodies level in serum. All values shown are mean ± s.e.m. (n = 9 mice in HDM+Vaccine group, n = 10 mice in PBS+Vaccine group and n = 10 mice in Trx+HDM group, missing sample due to failed collection of serum from the animal). *P < 0.05 in comparison to the PBS + Vacc group. Vacc: IL-33 vaccine, Trx: thioredoxin, HDM: house dust mite.

Chronic exposure of mice to HDM induced a HDM-specific IgG response (*P* < 0.05 compared to the PBS control). This response was not affected by vaccination against IL-33 (Fig 7).

### Vaccination against IL-33 attenuates the elevated levels of inflammatory cytokines present in lung homogenates from mice sensitized to HDM

**Fig 8:**
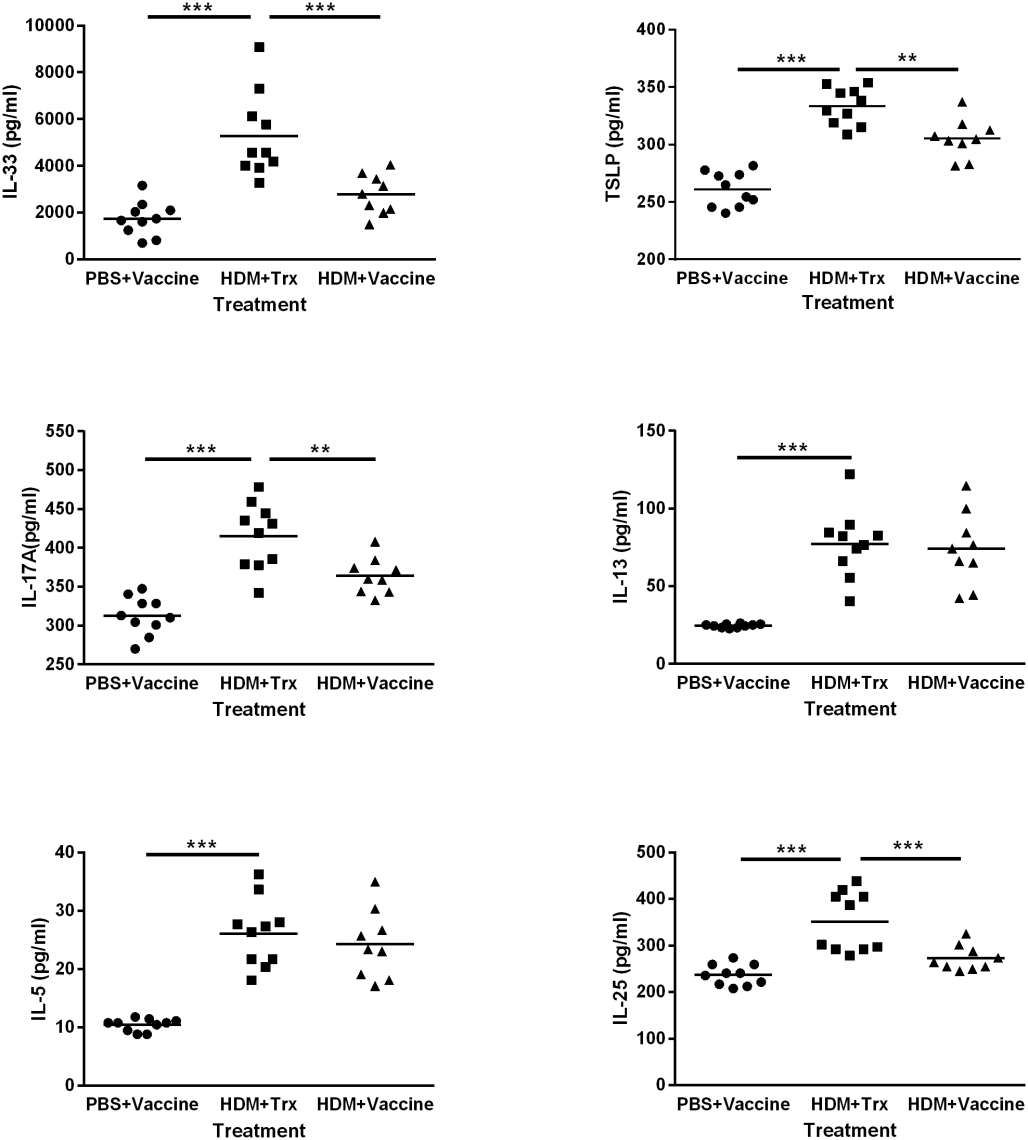
Vaccination against IL-33 attenuates the elevated levels of pro-inflammatory cytokine in lung homogenate supernatant from mice sensitized to house dust mite. The cytokines levels were measured using ELISA. All values shown are mean ± s.e.m. (n = 9 mice in HDM+Vaccine group, n = 10 in Trx+HDM group and n = 10 in PBS+Vaccine group, the lung sample from the animal failed to collect BALF was excluded). *** *P* < 0.001 in comparison to the HDM+Trx group. Vacc: IL-33 vaccine, Trx: thioredoxin, HDM: house dust mite.

In pulmonary homogenates isolated from HDM-sensitized mice the levels of all cytokines examined, i.e., IL-5 and IL-13 (Th2 cytokines), IL-17A (Th17) and IL-25, IL-33, and TSLP (produced by the airway epithelium) were potently elevated. Vaccination against IL-33 significantly inhibited these increases in IL-17A (*P* < 0.01), IL-25 (*P* < 0.001), IL-33 (*P* < 0.001), and TSLP (*P* < 0.01), without influencing the levels of IL-5 and IL-13 (Fig 8).

The levels of IL-5, IL-13 and IL-33 in BALF were below the detection limit of the ELISAs.

## Discussion

Our present findings demonstrate that vaccination against IL-33 elicits high titers of specific anti-IL-33 antibodies that markedly attenuate the development of airway inflammation and AHR, particularly within distal airways, in mice sensitized to HDM. These observations provide further support for the involvement of IL-33 in this inflammatory process and also indicate that IL-33 is a promising target for asthma intervention.

Vaccination has been one of the most important and effective strategies for combatting infectious diseases for almost 150 years. This impressive track record has awakened interest in applying this type of approach in other therapeutic contexts, such as asthma and other inflammatory conditions. However, self-tolerance makes it considerably more challenging to develop vaccines to endogenous than to bacterial or viral proteins. The self-antigen must be modified in order to trigger the immune system to elicit an antibody response, which can be achieved by insertion of dominant T cell epitopes into the antigen or by covalently coupling it to a non-self-protein to produce a fusion protein [3]. Here we used the small redox protein thioredoxin from E. coli as the non-self-component. It is advantageous to utilize small proteins in this connection to minimize the production of antibodies towards the fusion partner itself. This strategy for inducing anti-self-responses has been successful in several animal models including allergy, cancer and autoimmunity [23–25].

The second most important factor for successful vaccination against a self-structure is identification of a potent adjuvant. Indeed, the lack of such adjuvants for human use is probably the main reason why no vaccines of this type are yet in clinical use. As mentioned in the results, we have recently identified an adjuvant that works exceptionally well for vaccination against several endogenous targets in mice and rats [17, 23, 26, 27]. However, relevant questions concerning the safety of targeting an endogenous protein by vaccination have been raised. This issue has been investigated thoroughly in numerous previous studies, which show that the levels of antibodies raised against the self-antigen are dependent on both the modification of the antigen and the nature of the adjuvant. Since an unmodified self-antigen does not induce an antibody response even in the presence of a strong adjuvant and a modified self-antigen in the absence of a strong adjuvant does not evoke significant anti self-antibody titers, this strategy appears to be safe.

There has been much focus recently on the involvement of IL-33 in asthma and this cytokine as a potential therapeutic target [4, 15, 28]. To evaluate the efficacy of our IL-33 vaccine we employed an HDM-induced model of airway inflammation in mice with a long (42-day) sensitization and challenging period and a moderate dose of allergen (25 μg). This resulted in prominent AHR, with primarily eosinophilic inflammation, and potent elevations in cytokine levels, particularly those produced by the airway epithelium (IL-25, IL-33 and TSLP), but also the Th2-cytokines IL-5 and IL-13, as well as IL-17A.

For the measurement of AHR we applied the forced oscillation technique with a flexiVent, a small animal ventilator, which gives a detailed analysis of lung function. We found that the vaccination against IL-33 significantly inhibited the resistance in the conducting airways (*Rn*) (by approximately 50% compared to the Trx-control vaccine). In contrast, the tissue damping (*G*) was totally abolished. *G* reflects the parenchymal distortion that occurs when the airways constrict. A proportionally greater increase in *G* than *H* indicates the development of regional heterogeneities in the lung periphery [29]. Therefore, HDM-induced AHR in our murine model of asthma appears to affect both the proximal and distal airways and is highly dependent on IL-33.

Moreover, in our model the potent elevation in the number of eosinophils present in the BALF, and the lung inflammation evoked by HDM-sensitization were both inhibited by IL-33 vaccination. These findings are in agreement with previous reports that blocking IL-33 with mAb attenuates OVA-induced eosinophil infiltration [30, 31]. Vaccination against IL-33 also tended to reduce the goblet cell hyperplasia observed in HDM-exposed mice, although it did not reach significance compared to the group treated with the control vaccine.

IL-25, IL-33 and TSLP have been associated with asthma in a number of investigations, both in the clinic and in animal models, and reduction in their levels should have an important influence on AHR and airway inflammation. Similar to our results is the report that neutralization of TSLP or IL-25 inhibits airway inflammation and airway remodeling induced by chronic exposure to HDM [32, 33]. In the present study, the TSLP and IL-25 level in lung were significantly decreased by IL-33 vaccine. In addition we found that the HDM-induced production of IL-17A, another cytokine that may contribute to airway inflammation [34], was suppressed by our IL-33 vaccine. Cooperative and synergistic activities of IL-33 and TSLP have been reported in STAT5-mediated Th2 cytokine production and in the corticosteroid resistance during airway inflammation [35, 36]. IL- 33 was found to cooperate with IL-17A to exacerbate airway hyperresponsiveness in mice [37]. Thus, antagonism of cooperative effects by IL-33 blocking may also contribute to the inhibitory effects on airway hyperresponsiveness and airway inflammation by IL-33 vaccine. Similar to previous chronic HDM model of allergic asthma, increase of eosinophils in BALF and Th2 cytokines in lung tissue were also observed in our model[38]. Blockade of IL-33/ST2 has been shown to inhibit both airway hyperresponsiveness and Th2 cytokines in BALF in an OVA-induced allergic asthma model[39]; however, our data showed that IL-33 vaccine inhibited the HDM-induced airway hyperresponsiveness and eosinophilia, but not the increased lung Th2 cytokines. Gregory et al reported that when exposed to HDM extract for 5 days/week for up to 7 weeks in mice; the peak of airway resistance is at 24h post-final challenge, while the peak of eosinophil infiltration is at 8h post-final challenge and the peak of IL-5 and IL-13 production in lung is at 4h[40]. Therefore, one explanation to our results is that our read-out time point at 48h to acquire the highest response in HDM-induced airway hyperresponsiveness (based on our unpublished data) may occur later than the peak of IL-5 and IL-13 production in lung.

Il-33 has been shown to be of importance for the protection against numerous infections including parasites and cerebral malaria and also during non-viral hepatitis and for cardiovascular system repair. Caution should therefore be taken when modulating the *in vivo* levels of IL-33[41–45]. However the vaccine does not remove IL-33 but primarily act on excessive amounts of the cytokine and probably primarily act on IL-33 which is releases from the local tissue during inflammation. In addition, a very similar situation exists for TNF-alpha. This cytokine is one of the most central early inflammatory cytokines and still the use of anti-TNF binding proteins and humanized monoclonal is one of the most successful treatment strategies against several autoimmune conditions. A slightly increased incidence of mycobacterial infections is seen in some of the patients under anti-TNF treatment [46, 47]. So in line with these findings it is likely that also anti IL-33 treatment will be safe and serve as a successful therapy against several inflammatory conditions, including severe asthma and atopic dermatitis. However as stated above a careful monitoring of potential side effects need to be performed during all preclinical and clinical studies performed.

The vaccine developed and evaluated here can serve as a tool to elucidate the role of IL-33 in natural allergen-induced allergic lung inflammation, but our results also suggest that the vaccine could be further evaluated for therapeutic purposes. In summary, we have shown that a novel IL-33 vaccine elicits high titers of antibodies to endogenous IL-33 that are sustained and significantly inhibit HDM-induced AHR, airway inflammation and production of pro-inflammatory cytokines. These observations lend further support to the proposal that IL-33 plays a key role in the initial pathogenesis of asthma [28]. Exacerbations of asthma might in part be driven by IL-33, e.g., through its action on mast cells[48], why administration of this type of cytokine vaccine may prove to be an effective therapeutic tool for treating allergic asthma, as well as other phenotypes of asthma where IL-33 also are implicated[28]. The identification of a safe and soluble fusion antigen that together with a potent biodegradable adjuvant induces high antibody titers against native endogenous IL-33 also increases the chances for future clinical applications.
